# Human BLyS Facilitates Engraftment of Human PBL Derived B Cells in Immunodeficient Mice

**DOI:** 10.1371/journal.pone.0003192

**Published:** 2008-09-11

**Authors:** Madelyn R. Schmidt, Michael C. Appel, Lisa J. Giassi, Dale L. Greiner, Leonard D. Shultz, Robert T. Woodland

**Affiliations:** 1 Department of Molecular Genetics and Microbiology, University of Massachusetts Medical School, Worcester, Massachusetts, United States of America; 2 National Institutes of Health, The National Institute of Diabetes and Digestive and Kidney Diseases (NIDDK), Bethesda, Maryland, United States of America; 3 Department of Medicine, Division of Diabetes, University of Massachusetts Medical School, Worcester, Massachusetts, United States of America; 4 The Jackson Laboratory, Bar Harbor, Maine, United States of America; University of Miami, United States of America

## Abstract

The production of fully immunologically competent humanized mice engrafted with peripheral lymphocyte populations provides a model for in vivo testing of new vaccines, the durability of immunological memory and cancer therapies. This approach is limited, however, by the failure to efficiently engraft human B lymphocytes in immunodeficient mice. We hypothesized that this deficiency was due to the failure of the murine microenvironment to support human B cell survival. We report that while the human B lymphocyte survival factor, B lymphocyte stimulator (BLyS/BAFF) enhances the survival of human B cells ex vivo, murine BLyS has no such protective effect. Although human B cells bound both human and murine BLyS, nuclear accumulation of NF-κB p52, an indication of the induction of a protective anti-apoptotic response, following stimulation with human BLyS was more robust than that induced with murine BLyS suggesting a fundamental disparity in BLyS receptor signaling. Efficient engraftment of both human B and T lymphocytes in NOD *rag1^−/−^ Prf1^−/−^* immunodeficient mice treated with recombinant human BLyS is observed after adoptive transfer of human PBL relative to PBS treated controls. Human BLyS treated recipients had on average 40-fold higher levels of serum Ig than controls and mounted a de novo antibody response to the thymus-independent antigens in pneumovax vaccine. The data indicate that production of fully immunologically competent humanized mice from PBL can be markedly facilitated by providing human BLyS.

## Introduction

The development of a small animal model that reproducibly supports human lymphocyte development and/or peripheral lymphocyte survival and function should lead to improved treatment strategies for human tumors, autoimmune diseases and new vaccines. Immunodeficient mice, such as NOD-*Prkdc^scid^*, NOD-*rag1^−/−^ Prf1^−/−^*, NOD-*scid IL-2Rγ^−/−^*, Balb/c-*rag1^−/−^IL-2Rγ^−^* ^/*−*^ and H2^d^-*rag1^−/−^IL2Rγ^−/−^* have been used as recipients of human peripheral blood lymphocytes (PBL) or human hematopoietic stem cells (HSC) (reviews [1], [2]). Chimeric mice engrafted with human HSC can support B and T cell development and the survival of peripheral T and B cell populations [3]–[12], however lymphoid development following engraftment is slow (3–6 months) and is of varying efficiency even when the same HSC preparation is used to engraft multiple mice.

Conceptually, the engraftment of mature human peripheral blood lymphocytes (PBL) into immunodeficient murine recipients overcomes the need for long reconstitution times required when using human CD34^+^ cord blood derived HSC and should allow for a rapid assessment of immune responsiveness of diverse individuals, for example, young and aged humans. In addition, the potential to establish primary lymphoid tumors, such as leukemias and lymphomas, in a chimeric host would provide opportunities to investigate the efficacy of novel therapeutic strategies tailored to individual patients. T cell engraftment is frequently observed when PBL are transferred into NOD *rag2^−/−^ Prf1^−/−^* and NOD *rag2^−/−^IL-2rγ^−/−^* mice, whereas B cell homeostasis is abnormal. Mature B cells persist early after PBL transfer and can produce recall and polyclonal immunoglobulin (Ig) responses, however, these B cells are lost within 2 weeks and this loss is accelerated if CD4^+^ T cells are removed from the initial inoculum [13]. These data suggest that the murine environment may not provide the critical growth factors and/or signaling ligands necessary for B cell homeostasis.

Mature B cells are actively maintained in vivo by survival signals received through the B cell antigen receptor (BCR) and a receptor for the TNF family ligand B lymphocyte stimulator, BLyS, also known as BAFF, TALL-1, THANK, TNFSF13B and zTNF4 [14]–[17]. BLyS is a type II protein produced in both membrane-bound and soluble forms by stromal cells, macrophages, dendritic cells and neutrophils [18]. BLyS has two proposed mechanisms of action; to facilitate the differentiation of short-lived immature B cells into mature recirculating long-lived B cells, and to actively maintain mature B cells in the periphery by facilitating their survival through non-canonical NF-κB mediated signals [17], [19]–[21]. BLyS dependent survival signals are delivered through the BLyS receptor, BR3 (BAFF-R) that is expressed on late immature and mature peripheral B cells [18], [22]–[24]. Two other receptors for BLyS are also found on B cells, TACI and BCMA, and their expression is associated with different maturation and differentiation states [18], [25]. In humans, BLyS has been shown to be critical for B cell survival, the generation of lymphoid follicles and survival of plasmablasts formed from human memory B cells [24], [26], [27]. Overexpression of BLyS has been correlated with autoimmune diseases, such as systemic lupus erythematosus and rheumatoid arthritis [28]–[30], whereas lower levels of BLyS are associated with antibody immunodeficiency [31], [32]. In addition, some human lymphoid tumors (non-Hodgkins lymphoma and multiple myeloma) may produce BLyS as an autocrine growth factor promoting tumor survival [33]–[35].

Given the critical role of BLyS in normal B cell homeostasis, we proposed that the failure of efficient human B cell engraftment and survival in xenochimeras may be due to a BLyS deficiency. This failure could be due to species differences between human and murine BLyS that affect survival signaling. Consistent with this view, there is a single amino acid difference between the human and murine BLyS proteins in the portion of the molecule recognized by the BR3 receptor [18], [22], [23], [36].

In this report, we show human recombinant BLyS improves human peripheral blood B cell survival in vitro whereas murine BLyS is ineffective. Moreover, engraftment of both B and T cells is markedly enhanced in immunodeficient NOD *rag2^−/−^ Prf1^−/−^* mice supplemented with recombinant human BLyS. All B cell subpopulations are maintained, follicular-like structures develop and B cells secrete antibody and respond to challenge with thymus-independent pneumococcal antigens. Taken together, the data demonstrate the requirement for human BLyS for efficient engraftment of human B cells in immunodeficient mice.

## Results

### Human BLyS enhances human B cell survival in vitro

**Figure 1:**
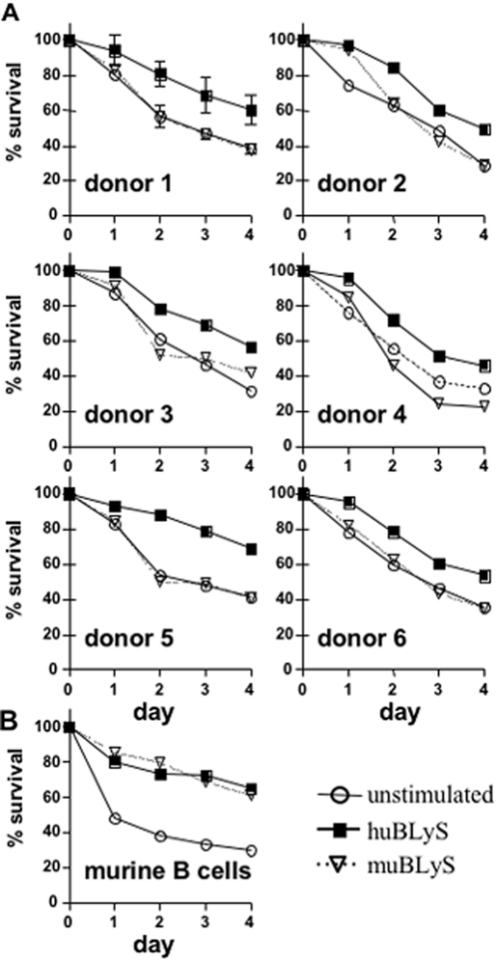
In vitro survival of CD19+ human B cells with human or murine BLyS. CD19^+^ B cells were purified from PBL by negative selection using RosetteSep kit and ficoll hypaque centrifugation. B cells were cultured for 4 days with 100 ng/ml of human or murine BLyS, cultures were resupplemented with BLyS on day 2. Viability was determined daily using cell counting with trypan blue and is represented as percentage of input cell number surviving. Donor 1 data is the average of 2 separate B cell preparations; donors 2–6 represent a single cell preparation. Statistical analysis for significance after 4 days in culture; huBLyS vs. muBLyS, p = 0.0041; huBLyS vs unstimulated, p = 0.0014; muBLyS vs. unstimulate, p = ns.

To establish possible species restrictions on BLyS dependent human B cell survival *in vitro*, CD19^+^ B cells from the PBL of normal human donors were cultured unstimulated or with human or murine BLyS and viability determined daily. The results from these determinations, Figure 1, clearly demonstrate that huBLyS enhances human B cell survival relative to either unstimulated or muBLyS supplemented cultures. Indeed, muBLyS provides no more survival advantage for human B cells than is seen in unstimulated cultures. Increasing the dose of muBLyS to 200 or 500 ng/ml did not improve human B cell survival (data not shown). Donor 1 was assayed on two separate occasions, 8 months apart, with similar results as indicated by the error bars in the graph. Statistical analysis of pooled data from 6 donors demonstrated the species dependence of BLyS mediated human B cell survival (at day 4 of culture: huBLyS vs. muBLyS, p = 0.0041; huBLyS vs unstimulated, p = 0.0014; muBLyS vs. unstimulated, p = ns). The species dependence of BLyS mediated human B cell survival was not observed for murine B cells, Figure 1B; both human and murine BLyS were equally effective at supporting murine B cell survival.

**Figure 2:**
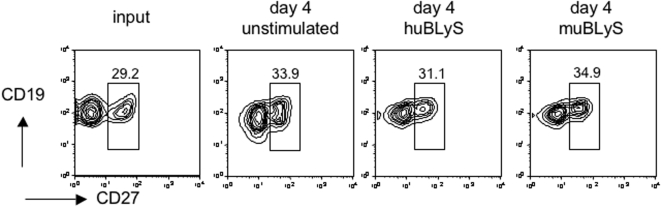
FACS analysis of B cell cultures. B cell populations were FACS analyzed for surface markers associated with resting B cells (CD45, CD19), memory cells (CD27), plasma cells (CD38) and kappa and lambda light chains on the day of isolation and after 4 days of culture either unstimulated or stimulated with human or murine BLyS. All samples were initially gated for lymphocytes by forward and side scatter. Data representative of 4 experiments.

To determine if huBLyS conferred a selective survival advantage to a particular subpopulation of PBL derived human B cells, input populations and cells remaining after 4 days of culture were compared by FACS using antibodies to CD19 (all B cells), CD27 (memory B), kappa and lambda light chains, CD10 (immature B), and CD38 (immature and plasma cells). Immature B cells (CD10^+^, CD27^−^, CD38^+^) represented 1–3% of input B cells from the various donors (our data and [37], [38]) and were undectable on day 4 analysis under all culture conditions. No other significant change in the character of the surviving B cell population relative to the input population as assessed by these markers was observed, Figure 2 data shown from CD19 and CD27 analysis.

### muBLyS binds to human B cells but does not mobilize NF-kB p52 as effectively as huBLyS

**Figure 3:**
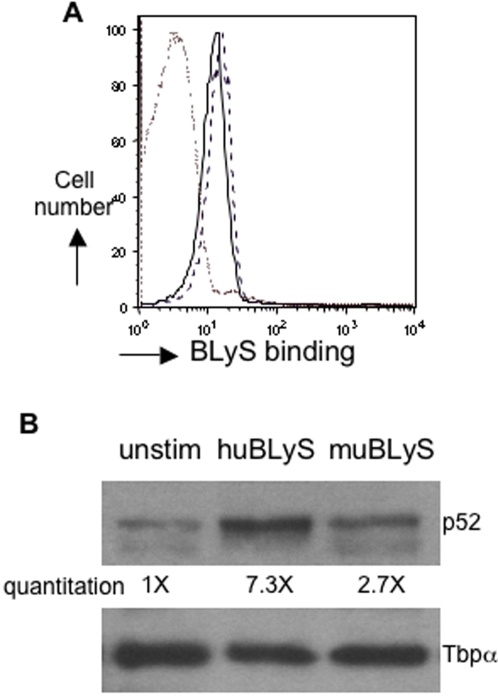
Nuclear localization of NF-κB p52. Purified CD19^+^ human B cells were A.) incubated with 100 ng/10^6^ cells of FLAG-huBLyS or FLAG-muBLyS followed by biotinylated anti-FLAG and strep-avidin PerCP on the day of isolation prior to FACS analysis. Control stain with anti-FLAG and PerCP (dash/dot line); huBLyS (solid line) and muBLyS (dark dashed line) B.) B cells were cultured unstimulated or with 100 ng/ml of human or murine BLyS for 48 hours. Cells were harvested, nuclear extracts prepared and Western blots prepared following protein separation on a 4–12% SDS gel. Blots were probed with anti-p52 antibody then stripped and reprobed with anti-TATAbpα antibody. Blots were developed by ECL. Data representative of 3 separate expts.

Murine BLyS has been shown to bind to human BLyS receptors [39], [40]. When assayed by FACS, we find that human B cells bind both hu and mu BLyS, tested at the optimal concentration used in our survival assays (100 ng) (Figure 3A) and also at lower concentrations (1 and 10 ng, data not shown). These studies did not determine which of the BLyS receptors were occupied. BLyS signaling induces both the canonical (NF-κB1) and non-canonical (NF-κB2) pathways; activation of the non-canonical NF-κB2 pathway is important for B cell survival [18], [19], [41]–[43]. Studies using murine B cells have demonstrated that nuclear localization of NF-κB p52 is sustained for at least 48–72 hours following BLyS stimulation (laboratory observations; [18], [19], [44]). Our in vitro survival data (Figure 1) shows that by 48 hours human B cell survival in cultures supplemented with muBLyS is significantly lower (p = 0.008) than cultures supplemented with huBLyS, accordingly, we choose this time point to initially examine differences in nuclear localization of NF-kB p52. Western analysis of nuclear extracts prepared from cells after 48 hours of culture demonstrates that nuclear accumulation of NF-kB p52 in B cells stimulated with huBLyS was 3-fold higher than in B cells cultured with muBLyS and 7-fold higher than unstimulated B cells (Figure 3B, representative example). An analysis of p52 nuclear localization in three separate B cell preparations showed huBLyS induced on averaged 7.1±1.6 fold increase over unstimulated B cells verses an average 2.4±0.9 fold increase with muBLyS. While, muBLyS stimulation does result in higher nuclear accumulation of p52 compared to unstimulated B cells, this is insufficient to support in vitro B cell survival.

To further investigate the notion that the amount of nuclear NF-κB p52 correlated with B cell survival, we cultured murine B cells with varying concentrations of huBLyS and assessed cell survival and p52 nuclear localization. We found the survival of murine B cells cultured with 1 ng/ml of huBLyS was similar to that observed in unstimulated cultures (figure S1A). In contrast, both 10 and 100 ng/ml of huBLyS supported murine B cell survival (figure 1 and figure S1A). After 48 hours of culture, nuclear extracts were made from all cultures, western blotted for NF-κB p52 and quantified. We observe that, similar to human B cells, murine B cells cultured with a sub-optimal huBLyS concentration mobilize low levels of p52 to the nucleus (supplemental figure 1B). It is noteworthy that the amount of nuclear p52 induced by the non-protective dose of huBLyS is similar to that seen with human cells cultured with 100 ng of muBLyS. Taken together, the data show a strong correlation between survival and the amount of p52 localized to the nucleus.

### Engraftment of human B cells in immunodeficient mice

**Figure 4:**
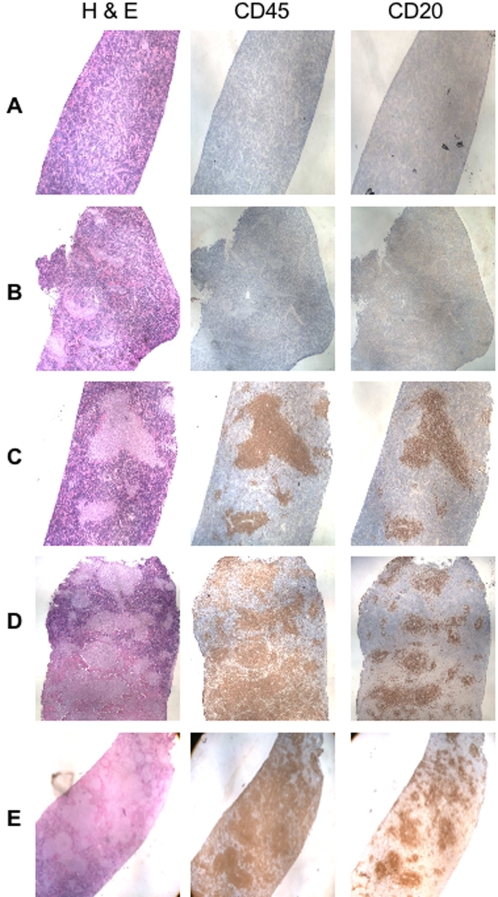
Immunohistology of in vivo PBL engraftment in NOD *rag2^−/−^ Prf1^−/−^* mice. On day of sacrifice, day 21 post PBL transfer, spleens were harvested and fixed for immunohistological analysis of B and T cell engraftment. Sections were visualized and photographed using a nikon microscope. All images were taken at 20× magnification. Rows A and B are PBS treated mice; C from mice treated 7 days with BLyS and, D and E from mice treated for 14 days with BLyS (10 ug/mouse/day). All mice were untreated days 14–21 prior to sacrifice. Serial sections were stained with hemotoxylin and eosin, anti-human CD45 or anti-human CD20. Data is representative of 6 experiments.

**Figure 5:**
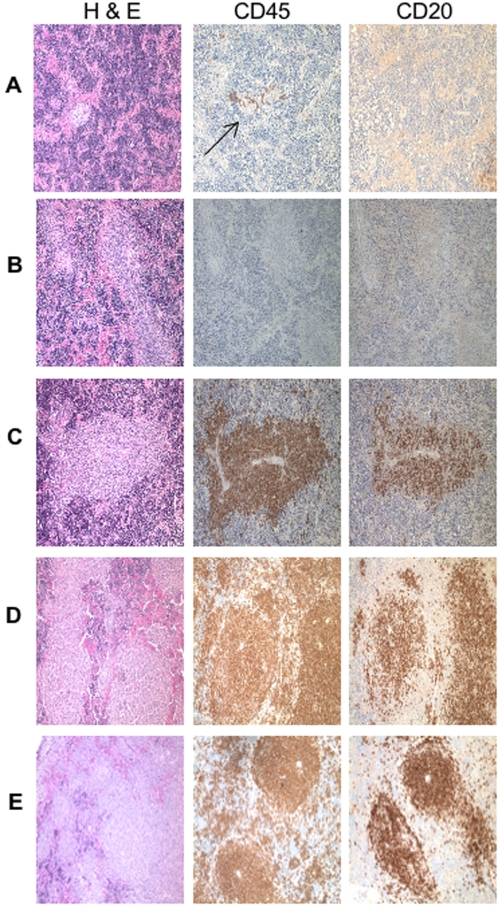
Immunohistology. Higher magnification images (100×) of portions of the sections shown in figure 3. Estimates of percentages of B cell engraftment are done at this magnification by comparing the percentage of the splenic section staining with anti-CD20.

To test whether huBLyS facilitated human B cell engraftment in immunodeficient mice, we transferred 20×10^6^ human PBL by intrasplenic injection into NOD *rag1^−/−^Ppf^−/−^* mice. Recipients were given human recombinant BLyS (10 ug/mouse/day) or PBS i.p. for 7–14 days and sacrificed for analysis on day 21. Spleen samples were assessed by immunohistochemical staining. Figure 4 shows representative serial sections of spleens from PBS or BLys treated mice stained with H&E, anti-human CD45 and anti-human CD20. In PBS treated animals, the H&E staining shows relatively uniform reticular tissue, with few scattered CD45^+^ cells and no obvious formation of typical splenic architecture; importantly, cells staining with anti-CD20 were not readily observed (Figure 4, A and B). In marked contrast, BLyS treated mice showed a different morphology by H&E staining. Numerous follicle-like collections of human lymphocytes were observed and these stained strongly with anti-CD45 (both T and B cells) and with anti-CD20 (B cells) antibodies (Figure 4, C–E, CD45 and CD20). Higher magnification of the serial sections shown in Figure 5 clearly demonstrates CD45^+^ cells in areas not staining with CD20 suggesting the presence of T cells (see below). For comparison, we estimate the extent of human B cell engraftment by averaging the area of CD20+ staining cells within a number of microscopic fields. Among mice treated for 14 days with human BLyS, B cells comprise 30–50% of the spleen section areas (Figures 4 and 5, D and E), whereas, mice receiving BLyS for only 7 days generally had smaller areas of B cell engraftment, approximately 20–25% of the spleen sections (Figures 4 and 5, C). We evaluated the possibility that the marked B cell engraftment we observed was the result of an EBV-mediated lymphoproliferative disorder, however, the B cells in BLyS treated recipients were negative for EBV when examined by EBER-1 *in situ* hybridization (data not shown). This data is consistent with previous observations suggesting that three-fold higher numbers of PBL or depletion of CD8^+^ T cells are required for EBV-mediated lymphoproliferation in engrafted immunodeficient mice [13].

**Figure 6:**
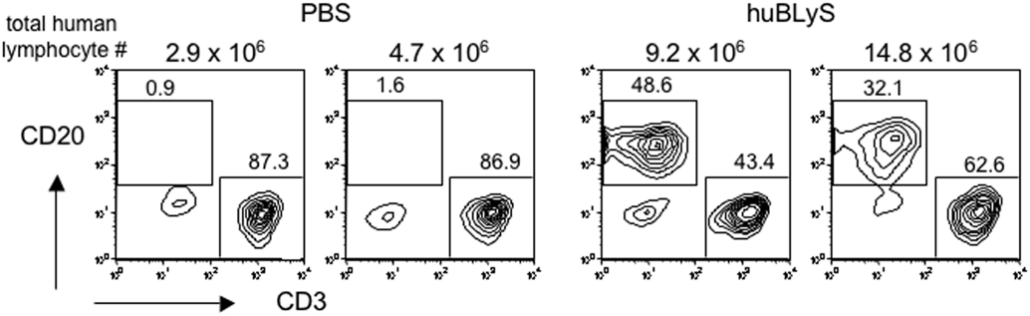
FACS analysis of collagenase disrupted engrafted spleens. One half of engrafted spleens were collagenase digested and then stained with anti-human CD45, CD20 and CD3 antibodies and analyzed by FACS. All samples were gated on live lymphocytes by forward and side scatter and then on CD45 positive cells. Data representative of 3 experiments. Total lymphocyte number = spleen cell count×percentage of CD45+ cells.

Whereas lymphocyte organization is revealed by immunohistology, more definitive quantitation of the human lymphocyte engraftment was assessed by flow cytometry. Spleens were divided in half upon harvest, with one half prepared for immunohistological analysis and the other prepared for FACS analysis by digestion with collagenase. Human lymphocyte yields (CD45^+^) from PBS treated recipients ranged between 0.27–5.8×10^6^ cells/spleen, whereas, significantly more cells were isolated from recipients treated with BLyS (7.7–20.2×10^6^ cells/spleen). The human B cell yields from the BLyS treated spleens was usually higher (4.1–9.5×10^6^ cells/spleen) than the number of input B cells (2×10^6^; 5–10% of total PBL) used to reconstitute the mice indicating that the engrafted cells had undergone proliferative expansion, perhaps by homeostatic proliferation induced by lymphocyte deficiency [45]–[47] or antigenic stimulation. Indeed, immunohistological staining of splenic sections with anti-Ki67, a marker for cells that have recently proliferated, was positive for almost all cells within the sections in BLyS-treated recipients whereas only scattered cells were positive in PBS-treated recipients (data not shown). Representative samples of collagenase disrupted spleens from separate experiments were analyzed by FACS (Figure 6) and showed that while T cells were present in some of the PBS treated mice (examples of positive engraftment shown with lymphocyte yield from test spleen), few, if any, B cells were detected in the same spleens. In contrast, both human B and T cells are readily detected in the huBLyS treated mice.

A summary of in vivo engraftment efficiency is shown in Table 1. Of the 16 mice treated with PBS, only 7 engrafted T cells and one of those 7 also engrafted B cells, overall 43% of the mice contained human lymphocytes. The PBS treated mice that were positive for human T or B cells had far fewer engrafted cells as determined by the sparse immunohistological staining; and reduced viable spleen cell yields. In comparison, 88% of the BLyS treated mice showed engraftment of both B and T cells with significantly higher numbers of donor splenocytes being isolated from these mice.

**Table 1:** Summary of in vivo engraftment data.

group	#mice	CD45 (T+B)*	CD20 (B)*	%positive
PBS	16	7	1	43
BLyS	33	29	29	88

Data from immunohistological analysis of splenic sections from NOD *rag1^−/−^ Prf1^−/−^* mice receiving 20×10^6^ PBL and then given 10 ug/mouse/day of huBLyS or PBS controls for 12–14 days and then sacrificed on day 21 post cell transfer. Sections were rated as positive if any CD45 or CD20 staining was observed at all.

* postitve sections as determined by immunohistological staining.

### Human BLyS is required for maintenance of the engrafted B cells

Human BLyS is required to initiate engraftment of B cells from human PBL in NOD *rag1^−/−^Ppf^−/−^* mice and we noted that mice receiving BLyS for only 7 days had lower amounts of B cells compared with mice receiving 14 days of treatment (20–25% vs 30–60%, determined by comparison of overall CD20 staining of histology sections, Table 2). To examine whether human B cells required the continued presence of huBLyS for in vivo survival as we would predict from murine B cell studies, a group of animals was treated for 7 days with huBLyS followed by injections of the soluble BLyS decoy receptor TACI-Ig protein (10 ug/mouse/day) for the next 7 days. TACI-Ig will bind both human and murine BLyS creating a BLyS deficient environment. Mice were sacrificed at day 21 and immunohistological analysis carried out on the spleens. We found that B cells were reduced when BLyS was withdrawn during PBS injections (about 2-fold lower) and were further reduced when mice received the BLyS decoy receptor (10-fold lower, Table 2, summary of histology results). Immunohistological analysis showed that T cells were still present (data not shown). Thus, maintenance of peripheral human B cell populations in reconstituted immunodeficient mice still require huBLyS.

**Table 2:** BLyS decoy receptor decreases B cell engraftment in NOD *rag1^−/−^ Prf1^−/−^* mice.

group	CD45	CD20	% B
BLyS 14 days	+	+	30–50
BLyS 7 days→PBS 7 days	+	+	20–25
BLyS 7 days→TACI 7 days	+	+/−	<5–10

In two experiments, NOD *rag1^−/−^ Prf1^−/−^* mice were given 20×10^6^ PBL were injected i.p. for 7 days with 10 ug/day/mouse of huBLyS, then divided into 3 groups of 3 mice each, the 3 groups received either PBS, 10 ug/day/mouse of huBLyS or 10 ug/day/mouse of TACI-Ig for 7 more days. Animals were sacrificed on day 21 and spleens analyzed by immunohistochemistry. Percentage of B cell engraftment was determined by analysis of the percentage of splenic sections staining with anti-CD20 and by FACS analysis of collagenase disrupted spleen samples.

### In vivo antibody production

To determine if the B cells in recipient mice were functional, we assessed human antibody production in mice treated with PBS or huBLyS for 14 days. NOD *rag1^−/−^Ppf^−/−^* mouse serum had undetectable levels of antibody. PBS treated recipients averaged 5 ug/ml of IgM and 60 ug/ml of IgG suggesting some early activation of transferred B cells prior to their loss from the animals. Recipients treated 14 days with BLyS had 40 fold more IgM, average 200 ug/ml, and 10 fold more IgG, 570 ug/ml consistent with the levels of B cell engraftment found (Table 3). To determine if antibody production was sensitive to BLyS depletion, mice were treated for 7 days with BLyS or 7 days with BLyS and then 7 days of TACI-Ig decoy receptor. Mice treated with TACI-Ig had approximately 8-fold lower amounts of IgM, 28 ug/ml, and 2-fold lower IgG compared to recipients receiving 14 days of BLyS treatment; consistent with TACI-Ig depletion of B cells and with the different half-lives of the antibody classes.

**Table 3:** Human serum Ig.

group	human IgM (ug/ml)	human IgG (ug/ml)
NOD rag^−/−^Pfp^−/−^ (n = 3)	ND*	ND
PBS (n = 6)	5.2±3.8	60.2±38.1
BLyS 7 days (n = 3)	26.1±4.6	438.7±89.9
BLyS 7 days→TACI 7 days (n = 3)	28.4±2.1	310.7±35.3
BLyS 14 days (n = 6)	200±44.5	572.5±22.7

Serum was collected at the time of sacrifice, day 21, and analyzed for human IgM and IgG synthesis from PBL engrafted mice treated for 14 days with PBS or BLyS, 7 days with BLyS, or 7 days with BLyS followed by 7 days of TACI-Ig. Data is representative of 3 experiments.

* ND, none detected, below the 3 ng sensitivity of the assays.

To assess the antigen responsiveness of transferred B cells, PBL recipients were immunized with pneumovax23, to test whether engrafted B cells could produce a de novo antibody response to a thymus-independent type 2 antigen. Recipient mice were vaccinated on the day of PBL transfer and serum collected at day 21 and analyzed with a serotype specific ELISA. Shown in Table 4 is the data for Streptococcus pneumoniae serotype 14, one of the 23 pneumococcal polysaccharide strains in the vaccine. Serum from PBL engrafted mice receiving PBS, huBLyS or PBS plus vaccine had a similar low quantity of anti-polysaccharide antibody, ranged between 0.7–1.4 ng/ml for IgM and 2.4–3.3 ng/ml for IgG. In striking contrast, recipients of huBLyS and vaccine averaged 10-fold higher amounts, 9 ng/ml IgM and 45 ng/ml IgG, of serotype 14 specific antibody. Similar levels of IgG and IgM were observed for pneumococcal serotype 4 (data not shown). These data demonstrate that the human B cells in PBL recipients can respond de novo to challenge with T cell independent antigens.

**Table 4:** Induction of a thymus-independent immune response in human PBL engrafted NOD *rag1^−/−^ Prf1^−/−^* mice.

Antibody Group	serotype 14 IgM	serotype 14 IgG
	ng/ml	ng/ml
PBS (n = 6)	0.68±0.16	2.88±0.35
PBS+pneumovax (n = 4)	0.72±0.12	2.42±0.23
huBLyS (n = 6)	1.35±0.41	3.30±0.51
huBLyS+pneumovax (n = 6)	9.02±3.77	45.6±20.4

PBL recipient mice were immunized with 20 ul/mouse pneumovax23 s.c. on the day of cell transfer. Serum was harvested on the day of sacrifice and assayed by ELISA for Streptococcus pneumoniae serotype 14 specific antibodies. Data representative of two experiments.

## Discussion

These experiments demonstrate the requirement for human BLyS to efficiently reconstitute human B cells in NOD *rag1^−/−^Ppf^−/−^* immunodeficient mice. A species specific BLyS restriction is demonstrated for human B cells in culture and for B cells transferred to murine hosts. The NOD *rag1^−/−^Ppf^−/−^* environment is not BLyS deficient, per se, as these mice readily support the engraftment of murine B cells. Species specificity in the action of survival and growth promoting cytokines is not novel to members of the TNF family; IL-2, IL-5, IL-6, and gamma interferon have all demonstrated species restrictions [18], [48]–[52]. Moreover, amino acid differences between murine and human BLyS in the BR3 receptor binding region and/or differences in the BLyS binding region of the BR3 receptor are known to have marked effects on survival signaling for human B cells [18], [22], [23], [53]. While we favor the notion that the basis for species restriction is in the interaction between BLyS and BR3, human B and T cells also express the TACI receptor for BLyS and differences in huBLyS and muBLyS binding to and signaling through TACI may also contribute to our results.

In vitro, human BLyS supported significantly better human B cell survival than did murine BLyS, which was indistinguishable from unsupplemented cultures. In contrast, human and murine BLyS were equally effective at promoting the in vitro survival of murine B cells. Concurrent with the survival effect, BLyS stimulation leads to a prolonged induction of NF-κB p100 processing and nuclear localization of NF-κB p52 (laboratory observations and [19], [44]). While freshly isolated human B cells bind FLAG-tagged murine BLyS as efficiently as human BLyS, p52 nuclear localization in human B cells induced by human BLyS was more robust than that induced by murine BLyS. This shows a dissociation between BLyS binding and survival signaling which may be the result of a species difference in the binding requirement for activation of BR3/TACI receptors on human B cells. It is noteworthy that in titration of human BLyS on murine B cells diminished B cell survival was correlated directly with the extent of p52 nuclear localization (supplemental figure 1). Our laboratory has recently defined the survival pathways induced by BLyS in murine B cells [54] and further experiments are in progress to define these pathways in human B cells.

Engraftment of human B cells is efficient in huBLyS treated NOD *rag1^−/−^Ppf^−/−^* recipients. Human BLyS is continually required to maintain the B cells over the 3 week period used in these studies since treatment with a BLyS decoy receptor, TACI-Ig, which depletes both human and murine BLyS from the animals, resulted in loss of B cells as assessed by immunohistological analysis and resulted in lower serum IgM and IgG compared with mice treated for 14 days with BLyS. Our engraftment data using huBLyS supplementation contrasts from that observed in other PBL xenochimera models where few, if any, human B cells are observed after cell transfer and those B cells do not survive beyond one week [10], [13], [55]–[60].

The B cells are functional as we find significantly higher amounts of human IgM and IgG in chimeras receiving huBLyS compared with PBS treated controls. Human Ig production could result from the homeostatic activation of naïve or memory B cells. Homeostatic B cell proliferation is seen when murine B cells are transferred into B cell deficient environments [45], [61], [62], however this mechanism has not been evaluate in xenochimeras. It is also possible that activation by xenoantigens can contribute to B cell activation and serum Ig. The antigen responsiveness of engrafted human B cells and the ability to induce a de novo immune response is demonstrated by the production of anti-pneumococcal antibodies following immunization with the thymus-independent antigens in pneumovax23. There was a 10–20 fold increase, respectively in IgM and IgG, in pneumococcal antibodies in BLyS treated recipients compared to BLyS treated and unimmunized and PBS treated immunized controls. This xenochimera model with enhanced B cell engraftment provides a unique opportunity to test vaccine responses using PBL samples from a variety of individuals, including neonates and the aged who frequently exhibit weak immune responsiveness.

Immunohistological analysis of recipient mice receiving only PBS revealed only scattered human T cells but these cells were readily identified by FACS analysis of collagenase digested spleens. It was noted, however, that there was a marked improvement of T cell engraftment with human BLyS supplementation. Both T and B cell yields from BLyS treated recipients was much higher than that observed with PBS treated recipients. Recent publications demonstrate that BLyS can act as a co-stimulator for T cells acting through BR3 and/or TACI [63]–[66]. T cells, like B cells, will under go homeostatic proliferation when introduced to a T cell deficient environment [45], [46], [67]–[69]. This proliferation causes induction of early activation markers, including expression of BR3 mRNA and protein on T cells [63]. Human T cells may also undergo activation to xenoantigens in the murine environment, again upregulating the BLyS receptor. In this regard, it is also possible that the human B cells themselves can present antigen to the T cells, thereby facilitating T cell expansion and survival. TACI-BLyS signaling in B cell-dendritic cell interactions have been shown to increase the expansion of antigen responsive CD8^+^ T cells [70] whereas BR3-BLyS costimulates CD4^+^ T cell alloresponses [66]. We did note that use of the TACI decoy receptor in vivo resulted in loss of both B and T cells, although B cells were affected to a greater degree. Taken together, these data suggest that human T cell engraftment may also be enhanced directly or indirectly by human BLyS treatment. Further studies using purified human T cells and human BLyS supplementation should address whether human BLyS acts directly on T cells to facilitate engraftment.

Since huBLyS enhances engraftment of both B and T cells, we considered the possibility that prolonged exposure to huBLyS may increase the rate of graft verses host disease (GVH) in the PBL recipient mice. No GVH was seen by observation or histological analysis at the 3 week time points assessed in these experiments even in recipients with the robust B and T cell engraftment, however, other investigators have found evidence of GVH by 4–6 weeks post-PBL engraftment in a variety of immunodeficient murine hosts [1], [71]. Regulatory T cells have recently been shown to express BLyS receptors and it is possible that huBLyS treated animals engraft sufficient regulatory cells to delay or prevent GVH [63], [65].

Our data suggest recombinant human BLyS has a significant enhancing effect on the engraftment of both T and B cell populations in PBL.

## Materials and Methods

### Human lymphocytes

Human blood was obtained from healthy volunteers and blood donors under signed consent in accordance with the Declaration of Helsinki and approval from the Institutional Review Board of the University of Massachusetts Medical School. Total human peripheral blood mononuclear cells (PBL) were purified by Ficoll gradient separation, quantified and viability assessed by trypan blue exclusion. Human CD19^+^ B cells were purified from PBL by negative selection using RosetteSep (StemCell Technologies, Vancouver BC, Canada).

### Murine lymphocytes

Murine B cells were prepared by anti-thy1.2 and complement treatment of splenocytes followed by purification of resting B cells using a step percoll gradient harvesting cells at the 60–70% interface [72].

### Mice

NOD/Cg-*Rag1^tm1Mom^Ppf^tm1Sclz^*/SzJ (abbreviated NOD *rag1^−/−^Ppf^−/−^*, stock # 004848) and C57BL/6 mice were obtained from Jackson Laboratories and NCI, respectively. Mice were housed at the University of Massachusetts Medical School under specific pathogen free conditions in accordance with federal and institutional IACUC guidelines. Immunodeficient mice received acidified (HCl; pH 2.8–3.2) water containing trimethoprim-sulfamethoxazole (Goldline Laboratories, Ft. Lauderdale, FL) ad librium for 7 consecutive days every other week.

### BLyS

Two sources of human BLyS were used in these experiments. Purified recombinant human BLyS (huBLyS, Human Genome Sciences, Rockville, MD 20850) or recombinant human FLAG-tagged BLyS (FL-BLyS) purified in our laboratory from transfected CHO cell supernant (cell line generously provided by Dr. Randolph Noelle, Dartmouth Univ. Lebanon, NH). Briefly, stably transfected CHO cells were grown in DMEM (7%FCS, 2 mM glutamine, 1× MEM non-essential amino acids, 10 units/ml penicillin and 10 ug/ml streptomycin) and supernatants collected. Supernatants were dialyzed against 50 mMTris pH 8.0, 50 mM NaCl, 0.02% sodium azide prior to running over an anti-FLAG M2 agarose column (Sigma-Aldrich Biochemicals). FL-BLyS was eluted with 0.1 M glycine HCl pH 3.0 and dialyzed against PBS, incubated with polymyxin B-agarose beads (Sigma-Aldrich, 1 ml of 50% suspension per 15 mls) for I hour, sterile filtered and quantitated by spectrophotometry (1 OD_280_ = 1.15 mg/ml). Purity was assessed by SDS-PAGE and Western blot. All human BLyS preparations tested negative for endotoxin and mycoplasma. Both huBLyS and FL-BLyS performed equivalently and were used interchangeably in these experiments. FLAG-tagged murine BLyS (muBLyS) (#522-052-C010) was obtained from Alexis biochemicals (Axxora, San Diego, CA 92121).

### Cell culture

Purified CD19^+^ B cells (94–98% pure by FACS analysis) were cultured at 5×10^6^ cells per ml in RPMI 1640 - complete media (CM: 10% FCS, 2 mM glutamine, 1× non-essential DMEM amino acids, 10 units/ml penicilin, 1 ug/ml streptomycin, 5×10^−5^ M 2-mercaptoethanol) alone or in the presence of 100 ng/ml of human or murine BLyS for 4 days. Preliminary experiments established the dose of BLyS sufficient for optimal cell survival: 1, 10, 100, 250 and 500 ng/ml were tested and no significant differences on cell survival were found at doses of 10 ng/ml or greater. BLyS was readded to the cultures on day 2. Cell survival was determined by counting daily using trypan blue exclusion for viability. Input populations and cells remaining after 4 days of culture were stained for human CD19, CD27, CD38, CD10, kappa and lambda light chains and analyzed by FACS.

### In vivo engraftment

In preparation for engraftment NOD *rag1^−/−^Ppf^−/−^* received a single i.p. injection of 1.0 mg of TMβ1 (anti-CD122) monoclonal antibody for depletion of NK cells and 10 ug of purified human recombinant BLyS (huBLyS). Recipients received 20×10^6^ PBL by intrasplenic injection followed by daily i.p. injection of 10 ug huBLyS or endotoxin free PBS for 7–14 days. Spleens were harvested 14–21 days post PBL transfer and processed for immunohistochemisry. In some expts, spleens were bisected and each half processed for either immunohistochemistry or FACS analysis.

### Decoy receptor

To deplete BLyS *in vivo*, groups of 3 mice that had previously received huBLyS for 7 days were given soluble TACI-Ig (10 ug i.p./mouse/day, Human Genome Sciences) for 7 subsequent days.

### Antibodies

Sources of anti-human antibodies used for FACS staining. Caltag: CD3-FITC, CD45-APC, CD45-PE, CD20-APC, CD19-APC, CD19-PE, IgD-FITC, CD38-PE; ebioscience: CD27-FITC, CD138-FITC and PE, CD10-biotin, kappa-biotin, lambda-biotin: BD Pharmingen: streptavidin-PerCP, IgM-APC, and CD45-PECy5.5. For immunohistochemistry BD Pharmingen: CD45, CD3, CD20, Ki67, and EBNA1.

### Immunohistochemistry

At sacrifice, spleens were fixed in 10% neutral buffered formalin, embedded in paraffin, and 5 um tissue sections were cut. Immunohistochemical staining was performed with human specific mABs (BD Pharmingen, San Diego, CA). Prior to staining sections were incubated in 0.1 M citrate buffer (pH 6.0) for 15 mins and then stained on a Dako (Carpinteria, CA) automated immunostainer using the EnVision (Dako) staining procedure. The sections were incubated with the EnVision plus Dual Link reagent (a polymer conjugated with goat anti-rabbit Ig or goat anti-mouse Ig and horseradish peroxidase) for 30 min. The sections were washed and reacted with 3-diaminobenzidine and hydrogen peroxide and counterstained with hemotoxylin for visualization by light microscopy.

### Flow cytometry

For in vitro experiments, purified PBL B cells were tested for purity and subpopulations characterized after initial isolation and 4 days of culture using CD45, CD19, CD20, CD27 and CD38 antibodies conjugated with APC, Fitc, PE or PerCp, described below. BLyS binding was determined by incubating 10^6^ purified B cells with 100 ng of murine or human FL-BLyS, followed by biotinylated anti-FLAG antibody (#F9291, SigmaAldrich) and strept-avidin PerCP (BD). FACS analysis of human lymphocyte engrafted mice was performed as follows: half of each recipient spleen was minced and incubated in 0.5ml HBSS containing 2.4 mg/ml collagenase XI (SigmaAldrich), 1 mg/ml DNAse I and 2% FCS for one hour at 37°C in a shaking water bath. Cell suspensions were then washed twice with CM, resuspended in 1 ml CM and incubated for one hour at 37°C in a CO_2_ incubator prior to FACS staining. Single cell suspensions were washed into FACS buffer (PBS, 3%FCS, 0.02% sodium azide) and10^6^ cells in 100 ul kept on ice for staining. Nonspecific staining was blocked by incubating cells with rabbit and mouse IgG (3ug) or 10 ul of C57BL/6 mouse serum for 10 mins on ice prior to adding fluorchrome-labeled antibodies. Cells were stained for 30–45 mins, washed and fixed with 2% paraformaldehyde prior to analysis. Engrafted cell numbers were determined from total cell counts isolated by collagenase multiplied by 2 (half of each spleen digested) and by the percentage of human CD45+ cells. Samples were analyzed using BD FACScalibur or FACSVantage machines and data analyzed using FlowJo software (Tree Star Inc., Ashland, OR).

### Western blotting

Purified CD19+ B cells were cultured unstimulated or with 100 ng/ml of mu FL-BLyS or hu FL-BLyS for 48 hours. Cells were harvested and nuclear and cytoplasmic extracts prepared as described [73]. Protein content was determined using Commassie protein assay reagent (Pierce Biotechnology, Rockland, IL) Samples were run on 4–12% NuPAGE MOPS gels (Invitrogen) and transferred to nitrocellulose membrane overnight. Membranes were blocked with 5% BSA, PBS, 0.2% Tween-20 buffer, anti-p100/p52 antibody (#4882, Cell Signaling Technology, Danvers, MA) or TATA binding protien (#AB818, Abcam, Cambridge Science Park, UK) as nuclear loading control added and incubated overnight at 4°C. Filters were washed with PBS+0.2%Tween-20, incubated with goat anti-rabbit HRP (Cell Signaling Technology), washed and developed using Amersham ECL plus detection system. Quantitation of signal was performed using a Molecular Dynamics Densitometer and BioRad Multi-analyst software.

### Determination of serum Ig

To assess polyclonal Ig production, sera were collected from cell recipients at the time of sacrifice and assayed for human Ig by ELISA. ELISA plates were coated overnight with 3 ug/ml F(ab′)_2_ goat anti-human IgG or IgM (Jackson Immunologicals) in PBS, 100 ul/well. Plates were washed with PBS, blocked for one hour with 0.5% BSA in PBS and serum dilutions added and incubated 1–2 hours. After washing, biotinylated anti-human kappa and/or lambda antibodies were added followed by washing and addition of streptavidin-HRP. The assay was developed with tetramethylbenzidine dihydrochloride (Sigma-Aldrich), stopped with sulfuric acid and read at 450 nm. Limits of detection were 3 ng/ml for IgM and 2 ng/ml for IgG.

### Immunizations

In two experiments, engrafted mice were immunized with pneumovax 23 (Merck, Whitehouse Station, NJ), 20 ul/mouse s.c. (containing 1 ug of each of 23 pneumococcal serotypes) on the day of cell transfer.

### Pneumovax ELISA

Immune responses to pneumovax23 were examined for 2 of the 23 pneumococcal serotypes, 4 and 14 (Danish strain designation) in the vaccine. ELISA analysis was performed according to WHO protocol (www.vaccine.uab.edu). Briefly, polystyrene ELISA plates were coated with 100 ul of 1 ug/ml serotype specific polysaccharides (type 4, ATCC #18-X and type 14, ATCC #23-X) for 5 hours at 37°C. Control human serum (89SF-2, US reference standard generously provided by Dr. Carl Frasch, CBER/FDA, Rockville, MD) with known concentrations of IgG and IgM to each serotype was used as a standard. Control serum and serial dilutions of unknowns were added to the plates, incubated overnight at room temperature. Plates were washed and incubated with anti-human IgG biotin or anti-human IgM biotin (#2040-08 and #2020-08, respectively, Southern Biotech, Birmingham, AL) for 2 hours, washed and strep-avidin-alkaline phosphatase (Southern Biotech) added for 1 hour. After washing the assay was developed with 1 mg/ml p-nitrophenyl phosphate in diethanolamine substrate for 2 hours, fixed with 3 M NaOH and read at OD 405 nm and 690 nm. Concentration determined as OD_405_-OD_690_ and read off the standard curve.

## Supporting Information

Figure S1 (0.15 MB TIF)
