# Supplementary figures and images for: Human BLyS Facilitates Engraftment of Human PBL Derived B Cells in Immunodeficient Mice
Source: PLoS One. 2008 Sep 11;3(9):e3192. doi: 10.1371/journal.pone.0003192 (PMC2527131; doi:10.1371/journal.pone.0003192)

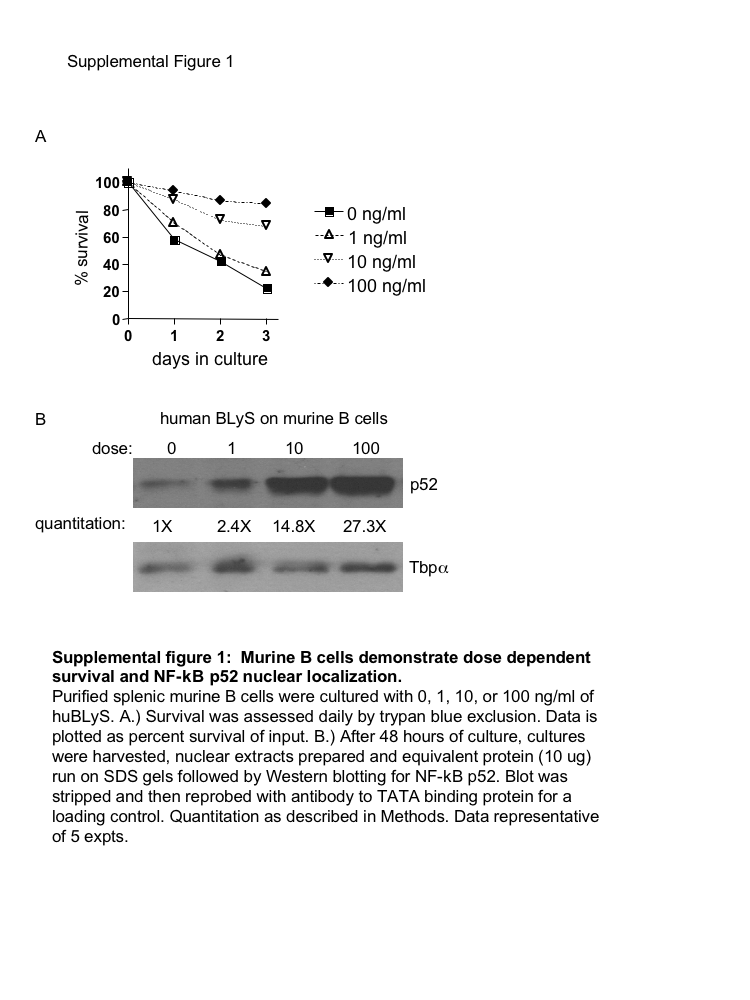

Supplement: Figure S1 — (0.15 MB TIF) [file pone.0003192.s001.tif]
